# Introspective accuracy for substance use across a year of treatment for first episode psychosis

**DOI:** 10.1016/j.scog.2021.100200

**Published:** 2021-05-27

**Authors:** Joshua E. Mervis, Jamie Fischer, Samuel E. Cooper, Andrew C. Deckert, Paul H. Lysaker, Angus W. MacDonald, Piper Meyer-Kalos

**Affiliations:** a University of Minnesota, Department of Psychology, United States of America; b University of Minnesota, School of Social Work, United States of America; c University of Minnesota Medical Center, Department of Psychiatry and Behavioral Sciences, United States of America; d University of Texas at Austin, Department of Psychiatry and Behavioral Sciences, United States of America; e Richard L. Roudebush VA Medical Center, United States of America; f Indiana University School of Medicine, Department of Psychiatry, United States of America

**Keywords:** Introspective accuracy, First episode psychosis, Substance use, Insight

## Abstract

Substance use exacerbates psychosis, mania, depression, and poor functioning in people with first episodes of psychosis (FEP) and is associated with poor treatment outcomes, even when it does not reach the level of a formal disorder. Impaired insight and substance use are common issues that may interfere with treatment outcomes among people experiencing FEP, yet both are treatable. Improvements in these domains are associated with better outcomes. Low insight could increase risk for substance use by impairing the ability to self-appraise and assess consequences. Introspective accuracy (IA) is understudied in this area and is one way of considering self-appraisal. This study is an archival review using data collected from NAVIGATE, a coordinated specialty care program treating people with FEP. IA was operationalized as the difference between clinician and client ratings of substance use. We tested whether IA changed over one year of treatment and whether those changes occurred alongside changes in symptoms and illness self-management. No changes in IA were detected in relation to illness self-management. Changes in IA for substance use occurred midway through treatment—individuals with greater symptom remission had more overconfident IA. Prior research on insight has shown a paradox where greater insight accompanies more symptoms. However, past research has also shown a relationship between IA and functional outcomes, like illness self-management, and that overconfidence in one domain can positively bias clinician ratings in another. Our findings suggest either a positive bias for ratings associated with overconfident IA or an insight paradox type effect.

## Highlights

• Introspective accuracy is a form of insight that can be applied to early psychosis.
• Introspective accuracy for substance use paradoxically interacted with symptoms
• Introspective accuracy for substance use was unrelated to illness self-management.

## 1 Introduction

Substance use can impede progress in psychological interventions, and it is important to understand its role in outcomes for mental health treatment of co-occurring substance use (Kay-Lambkin et al., 2011) and schizophrenia (Czobor et al., 2015). Despite this, substance use is treatable in people with serious mental illness (Barrowclough et al., 2001; Daley et al., 1998; Smeerdijk et al., 2012), and is a common treatment target in First Episode Psychosis (FEP) (Addington et al., 2013; Heinssen et al., 2014) even when it does not reach the level of a formal disorder. Understanding constructs related to substance use in the context of treatment could also provide an opportunity to refine treatment approaches in early intervention.

In the context of mental illness, insight is the degree of lucidity towards experiences related to illness (Pick, 1882) and related inferences about oneself (Lewis, 1934). Much like substance use, impairments in insight are of relevance to intervention research in people with psychosis. These impairments can impact treatment in similar ways—poor insight is associated with poor treatment engagement and outcomes (Elowe et al., 2020; Garcia-Cabeza et al., 2018; Lysaker et al., 1994, Lysaker et al., 2013; Schwartz, 1998). Limited research has shown that treating insight in schizophrenia yields improvements in substance use outcomes (James et al., 2018). Several studies indicate that both subjective and objective improvements in insight are possible within the first two to three years of treatment following a first episode of psychosis (Phahladira et al., 2019; Saeedi et al., 2007; Vohs et al., 2018). Other research in this population indicates these improvements mitigate depression secondary to substance use (Elowe et al., 2020). However, some research suggests an insight paradox, wherein better insight is associated with worse outcomes. For example, better insight is linked to greater depression (Lysaker et al., 2007; Ramu et al., 2019; Tariku et al., 2019), but this is inconsistently observed (Arraras et al., 2019; Konsztowicz and Lepage, 2019). Relatedly, increased suicidality and attempts are sometimes (Ozturk et al., 2018; Villa et al., 2018), but not always (Ayesa-Arriola et al., 2018) associated with better insight.

Researchers have noted that the development of insight via the mechanism of self-reflection is a useful target in treatment models for people with schizophrenia and is therefore useful to measure (Lysaker et al., 2011; Lysaker and Dimaggio, 2014; Moritz and Lysaker, 2018). While not specifically investigated as insight around substance use, there appear to be deficits in the self-appraisal of substance use and related consequences in people with FEP. A meta-analysis of psychosis-related outcomes across the lifespan found that younger people were more likely to use some form of substance and have more severe positive symptoms (Large et al., 2014). Approximately 50% of individuals with FEP have or had a substance use disorder, which increases symptom severity and stigma (Brunette et al., 2018). Using cannabis as an example, a meta-analysis of the prevalence of use in people with FEP found that around a third reported any cannabis use (Myles et al., 2016). In contrast to other research (Brunette et al., 2018), the same study also found that use decreased at follow up ten years after the first episode. A large longitudinal study of FEP found that cannabis use increased the overall severity of psychotic symptoms, mania, and depression, as well as low psychosocial functioning (Seddon et al., 2016). Individuals who kept using cannabis over the first year after an initial episode had more severe symptoms and poorer psychosocial outcomes than others in the sample. These findings are in line with other longitudinal population studies and meta-analyses finding cannabis use associated with more severe outcomes across the illness course, regardless of reaching the level of a formal disorder (Degenhardt and Hall, 2006; Henquet et al., 2004; Large et al., 2011; Semple et al., 2005; van Os et al., 2002). Others found the same association for substance use broadly (Large et al., 2014). Illustrating all of the above, individuals with FEP who continued using cannabis past the first year of treatment had poorer insight and treatment outcomes over the total three years of treatment (Elowe et al., 2020).

Researchers have noted that understanding insight in the context of substance use could be important to treatment outcomes, but measuring the construct is a challenge (Raftery et al., 2019). Only one instrument measures insight related to substance use in particular, which is limited to alcohol use and self-report (Raftery et al., 2020). There is no universal measure of a person's self-appraisal of substance use that incorporates more than self-report. One solution lies in how self-appraisal can be operationalized as *introspective accuracy* (IA) (Nisbett and Wilson, 1977), using one's subjective evaluations as comparison to objective ones. Examples of objective anchors include interview, informant, or performance-based measures, with IA representing the magnitude of the difference between subjective and objective ratings.

IA has been recently studied in schizophrenia research to assess targets that are highly relevant to recovery like functioning, social cognition, and neurocognition, generally finding poor IA is associated with poor outcomes (Halverson et al., 2019; Pinkham et al., 2018b; Silberstein and Harvey, 2019; Springfield and Pinkham, 2020). IA regarding substance use is likely to play a role in functional recovery through understanding one's potential to get well (Harvey and Pinkham, 2015; Silberstein and Harvey, 2019). This in turn could increase and individual's engagement with their treatment through identifying goals or celebrating accomplishments. However, to date we are unaware of any empirical evidence suggesting that changes are in fact related to recovery outcomes such as symptom remission and self-management of illness. To test this, we used archival data in existing local medical databases for people treated in the NAVIGATE model over one year (Kane et al., 2016; Mueser et al., 2015). We operationalized IA for substance use as the difference between clinician and client ratings of substance use within a treatment setting. We then examined whether we could detect changes in IA over time and whether those changes were related to clinician ratings of symptom remission and illness management.

While this study is exploratory in nature, there are general hypotheses about the clinical milieu. We hypothesized that IA for substance use would change over time along with improvements in measures of recovery. People with stronger recovery ratings would demonstrate better IA through the course of treatment. Because individuals with FEP tend to span a wide age range in early adulthood and age may be a confound, we also controlled for the effect of age in our analysis.

## 2 Methods

This study was approved by the local institutional review board and determined non-human subjects research. Individuals were assessed at three time points using one formal clinician-rated assessment measure and a corresponding client-rated assessment measure. Both measures were given at all timepoints by the individual and their program therapist. Participants were diagnosed by their program therapists, including psychologists, social workers, and marriage and family therapists. Diagnoses were not limited to a primary diagnosis, so individual had multiple diagnoses in some cases (e.g., schizophreniform and cannabis use disorder). However, we report the primary diagnosis.

### 2.1 Sampling procedure

We accessed 329 deidentified records of individuals in treatment as part of a NAVIGATE program to treat people with FEP. The NAVIGATE program used admission criteria from the effectiveness study (Kane et al., 2016): age 15–40, as well as a diagnosis of schizophrenia, schizoaffective disorder, schizophreniform disorder, brief psychotic disorder, or unspecified psychotic disorder. Individuals were included in the analysis if they had no more than 3 items missing on the Illness Management and Recovery Scale, which is described below. More specifically, missing data could not all be on the same subscale, and none could be missing for the items used to calculate IA. We found 44 records without missing data over the first twelve months of treatment, and an additional 21 records with missing data and imputed median values. In total, we removed 264 records for incomplete data and retained those with complete data for the baseline, six months, and twelve-month assessments. The characteristics of the final sample (N = 65) are detailed below in Table 1.

**Table 1:** Demographics and clinical characteristics of the sample (n = 65).

Variable	Mean (SD) or % (N)
Age (years)	21.22 (4.27), range: 15–34.75
Sex (men)	78 (47)
Race	
White	48 (32)
Ethnoracial minority^a^	52 (33)
Hispanic/Latinx	32 (21)
Education	
Completed high school/GED	23 (15)
Some high school	26 (17)
Some college	29 (19)
Other	22 (14)
Intake diagnosis	
Psychotic disorder^b^	75 (49)
Primary diagnosis missing^c^	26 (16)

Note: All measurements depicted above are at baseline.

a American Indian and Alaska Native (3%), Asian (2%), Black or African American (26%), Biracial (11%), Other (8%), Unspecified (2%).

b Unspecified Psychotic Disorder (N = 21), Schizophrenia (N = 16), Schizoaffective Disorder (N = 4), Schizophreniform (N = 6), Substance Induced Psychotic Disorder (N = 2).

c Primary Diagnosis Missing (N = 16).

### 2.2 Assessments

#### 2.2.1 The Illness Management and Recovery Scales (IMR-Client and IMR-Clinician)

The IMR Client and IMR Clinician Scales are 15-item standardized self-report scales used to measure perceptions of life and treatment goals, psychiatric symptoms, recovery, supporter involvement, and role functioning for the past 90 days (Färdig et al., 2011; Salyers et al., 2007). The client rating scale is self-reported, and the clinician rating scale is observer reported. Each question is behaviorally anchored and uses a 5-point Likert-type rating, and the items on this scale can be interpreted separately or as an overall total. These behavioral anchors are similar for both clients and clinicians; for example, an item might ask both to separately consider the client's progress in relapse prevention planning in case of symptom exacerbations, to reduce the likelihood that a higher level of care is needed. For clinicians, these ratings refer to the client's response to and understanding of pros and cons of substance use, as well as associated problems. They also refer to developing support structures to prevent use, in addition to risk, social, and emotional management related to use, to prevent use relapse. For clients, these ratings refer to their ability to understand and execute the above behaviors.

In a study of over ten-thousand individuals, the IMR scales showed good internal consistency, criterion validity, and construct validity (Sklar et al., 2012). Factor loadings for items included by Sklar and colleagues had good construct validity in their sample. Rather than use the total score or individual item approach, the current study uses subscales based on these studies that supported a three-factor model of the IMR scales, as this was a better fitting solution compared to a single factor. Factors were Illness Recovery (four items), Illness Management (five items), and Substance Use (two items) subscales, using the item configurations from Sklar and colleagues. The recovery subscale is comprised of the symptom distress, impairment of functioning, relapse of symptoms, and coping items. The illness management subscale is comprised of progress towards personal goals, knowledge, contact with people outside of family, relapse prevention planning, and involvement with self-help activities. The substance use subscale is comprised of the impairment of functioning through alcohol use and drug use items. For brevity, item wording is not included here, but the IMR scales are available in full in the original validation article (Salyers et al., 2007, p. 475–479).

The factor analysis producing these subscales did not retain four items from the original scale because they did not load on any factors: involvement of family and friends in treatment, time in structured roles, psychiatric hospitalizations, and using medication effectively. Since this study tested an operationalization using the constructs of the IMR Scales and is not a test of the scales themselves, the term “illness self-management” refers to the IMR Illness Management subscale, as well as the general construct of managing an illness. Additionally, the term “symptom remission” refers to the IMR Illness Recovery subscale, as well as the general construct of symptom remission.

### 2.3 Introspective accuracy for substance use

In our review of the literature, published operationalizations of insight related to substance use consisted of whether someone “admits” or acknowledges, or agrees with raters that one has problematic use. A conceptual challenge with this is that a binary response variable assumes that the raters are correct without providing a magnitude of disagreement, if present. We subsequently operationalized insight as IA for Substance Use, measured as the difference score between clinician and client ratings of substance on the IMR Scales, using the subscales described above. This is a novel approach for measuring substance use in clients. We used this operationalization because it is common for clinicians to assess substance use with their clients and ask them to assess their own substance use. Given that these are two different vantage points, it is reasonable that there could be dissonance between the two scores, with clinically meaningful variance potentially reflected in that difference. First, each subscale was calculated by adding and averaging the corresponding individual items. Following this, the deviation score was scaled so that higher values on this item reflected better underestimating of progress, whereas lower scores reflected overestimation. Scores close to zero reflect more “accurate” estimations of insight on the part of those with FEP. While this operationalization inevitably creates challenges in the interpretation of analyses, it preserves the direction of discrepancies, a distinction from using another operationalization that focuses only on the magnitude of the difference, like an absolute value. This operationalization also allowed for a continuous distribution as opposed to characterizing the data in groups reflecting overestimating, underestimating, and accurate estimating. A conceptual strength of our operationalization was that it affords both continuous and qualitatively different interpretations of the data.

### 2.4 Planned analysis

People with first episode psychosis and their clinicians rated these scales at each measurement time point. IA for substance use, the focus of this study, was calculated based on the operationalizations noted above. Time points were at approximate intervals of baseline, six months, and twelve months, but were coded as a factor since the exact number of weeks between assessment timepoints differed from person to person, and some clients spent time away from the treatment program so did not receive the same “dose” of treatment. All variables were standardized as Z-scores before analysis.

All analysis was conducted in R Studio (Core Team, 2015). Separate planned linear mixed effects regressions were as follows: 1) IA for substance use predicted by the interaction of timepoint and illness management, controlling for age, and 2) IA for substance use predicted by the interaction of timepoint and symptom remission, controlling for age. We used the *lme4* library (Bates et al., 2007) to estimate all linear mixed effects models. For these models, a random intercept at the individual participant level was the only random effect included. Timepoint was entered as a categorical fixed-effect predictor (with baseline entered as the reference condition), and illness management. Symptom remission ratings were entered as continuous fixed-effect predictors in their respective models. Each model also contained an interaction term, consisting of timepoint and the continuous predictor of interest.

We used the *parameters* (Lüdecke et al., 2020) library to extract conditional and marginal R^2^ (with conditional R^2^ analogous to R^2^ for standard linear models, and marginal R^2^ the estimate of the explanatory power of only the fixed-effects in the model). Standardized parameters were obtained by fitting the model on a standardized version of the dataset. 95% Confidence Intervals (CIs) and *p*-values were computed using the Wald approximation.

## 3 Results

The baseline characteristics of variables used in our analysis are shown below in Table 2.

**Table 2:** Baseline values for unstandardized variables.

Variable	Mean (SD)
Introspective accuracy for substance use	−0.60 (0.95)
Clinician-Rated Illness Management	2.01 (0.66)
Clinician-Rated Symptom Remission	2.73 (0.61)

Note: Higher scores on the clinician-rated variables and lower scores on the insight variable are more desirable. Subscales are those produced from (Sklar et al., 2012). Subscale scores are an average of items on those scales.

Our hypotheses were that we could detect changes in IA over time and that those changes would be related to changes in recovery, such that people who had stronger recoveries would have better IA. One of our recovery models addressed whether individuals with better illness management had better IA over time. The illness management model's total explanatory power was moderate (conditional R^2^ = 0.23) and the part related to the fixed effects alone (marginal R^2^) is 0.08. In terms of main variables of interest, the interaction between timepoint and illness management yielded non-significant tests, both for the change in IA from baseline to 6 month timepoint, (std *β* = −0.23, 95% CI [−0.65, 0.18], *t*(186) = −1.10, *p* = 0.273), as well as from baseline to 12 month timepoint, (std *β* = −0.20, 95% CI [−0.61, 0.22], *t*(186) = −0.93, *p* = 0.355). See Table 3 for complete model parameter estimates and fit statistics.

**Table 3:** Illness self-management mixed effects model.

	Introspective accuracy for substance use^a^				
Predictors	*β*	std. error	CI	Statistic	*p*
Intercept	−0.14	0.18	−0.49–0.21	−0.79	0.432
Age at intake	−0.04	0.08	−0.19–0.12	−0.46	0.645
Illness *self-*management	0.28	0.18	−0.07–0.63	1.57	0.117
6-Month timepoint (vs. baseline)	0.34	0.20	−0.06–0.74	1.66	0.098
12-Month timepoint (vs. baseline)	0.23	0.22	−0.20–0.65	1.06	0.290

Illness *self*-management × timepoint interaction terms					
Illness *self-*management: 6-month timepoint (vs. baseline)	−0.23	0.21	−0.65–0.18	−1.10	0.273
Illness *self-*management: 12-month timepoint (vs. baseline)	−0.20	0.21	−0.61–0.22	−0.93	0.355


Random effects	
σ^2^	0.77
τ_00 Individual_	0.15
ICC	0.16
N _Individual_	65
Observations	195
Marginal R^2^/conditional R^2^	0.075/0.226
AIC	549.707

Note: AIC = Akaike information criterion; ICC = intraclass correlation coefficient.

a Introspective accuracy scores computed as clinician rating minus self-report score.

Another of our recovery models addressed whether individuals with better symptom remission had better IA over time. The symptom remission model's total explanatory power was substantial (conditional R^2^ = 0.28) and the part related to the fixed effects alone (marginal R^2^) was 0.10. In terms of main variables of interest, the interaction between timepoint and illness management yielded a significant test for the change in IA from baseline to 6 months on symptom remission (std *β* = −0.45, 95% CI [−0.85, −0.06], *t*(186) = −2.27, *p* < 0.05). The interaction effect for the change in IA from baseline to 12 months on symptom remission was non-significant (std *β* = −0.15, 95% CI [−0.55, 0.25], *t*(186) = −0.73, *p* = 0.467). See Table 4 for complete model parameter and fit statistics.

**Table 4:** Illness recovery mixed effects model.

	Introspective accuracy for substance use^a^				
Predictors	*β*	std. error	CI	Statistic	*p*
Intercept	−0.01	0.18	−0.36–0.34	−0.08	0.939
Age at intake	−0.01	0.08	−0.17–0.15	−0.11	0.916
Symptom remission	0.39	0.16	0.08–0.70	2.47	**0.014**
6-Month timepoint (vs. baseline)	0.25	0.21	−0.16–0.65	1.18	0.239
12-Month timepoint (vs. baseline)	0.03	0.22	−0.40–0.45	0.13	0.899

Symptom remission × timepoint interaction terms					
Symptom remission: 6-month timepoint (vs. baseline)	−0.45	0.20	−0.85 to −0.06	−2.27	**0.023**
Symptom remission: 12-month timepoint (vs. baseline)	−0.15	0.20	−0.55–0.25	−0.73	0.467


Random effects	
σ^2^	0.72
τ_00 Individual_	0.18
ICC	0.20
N _Individual_	65
Observations	195
Marginal R^2^/conditional R^2^	0.101/0.281
AIC	543.706

Note: AIC = Akaike information criterion; ICC = intraclass correlation coefficient.

Bold values indicates statistically significant at P < .05.

a Introspective accuracy scores computed as clinician rating minus self-report score.

## 4 Discussion

IA for substance use refers to an individual's self-appraisal of their current usage benchmarked against an objective rating of their current usage, in this study's case that of their clinician. The present study's primary aim was to test an operationalization of IA for substance use and detect changes over time corresponding to illness self-management and symptom remission among people in treatment for FEP. We hypothesized that people with stronger recovery ratings would have better IA over time. Contrary to expectations, we found no effect for fluctuations in IA for substance use related to illness management. That is, over a year of treatment whether an individual improved, declined, or stayed the same with regard to the management of their illness, there was no impact on their IA. We found a mid-treatment interaction for IA for substance use and symptom remission, such that greater symptom remission was related to lower IA at the 6-month assessment.

While this finding was surprising, there are several possible explanations. One explanation for the interaction at 6 months but not 12 months is that people who will respond to treatment have responded by that point, creating variability in scores and a subsequently larger, detectable effect. Another possibility is there that are genuinely negative effects of better IA, as noted in recent work on IA and functioning (Olsson et al., 2019) and subjective neurocognitive complaints (Raffard et al., 2020). As to why better symptom control could accompany poorer IA, evidence suggests that the depressive clinical insight paradox in schizophrenia (Davis et al., 2020; Lysaker et al., 2018, Lysaker et al., 2007; Vohs et al., 2016) applies across domains of IA (Harvey et al., 2017; Jones et al., 2020; Siu et al., 2015). Consistent with the specific findings of the present study, other work showed that individuals who overestimated their abilities (lower values for IA) had the least depression (Harvey et al., 2019).

One of the weaknesses of how we operationalized IA is that we assumed that clinicians' ratings of their client's substance use would be an accurate anchor. It is possible that the lack of an effect for illness management represents a bias created by our operationalization. However, other work using this operationalization for other domains shows consistent positive associations between better IA and better functioning (Gould et al., 2015; Olsson et al., 2019; Pinkham et al., 2018a; Silberstein et al., 2018), which is similar to illness management as measured here. The question of which particular rater is used as an anchor is also relevant, as past work has found relationships between IA and different variables based on usage of clinician or caregiver ratings (Nishida et al., 2018).

There are limitations to this naturalistic study based on an archival review. First, the design has no blinding, control group, randomization, or standardization of raters. Second, the sample size is small. Third, since there was no control over instruments, none specifically assessed clinical or cognitive insight, which are the most studied insight constructs (Lincoln et al., 2007; Lysaker et al., 2018; Vohs et al., 2016). Additionally, we did not have access to information on raters and thereby could not include it as an additional random effect in our modeling. We also did not have access to high contact informants (e.g., partner, parent), which could have yielded a more robust measure of IA. In fact, research has found different relationships between IA derived from high contact informants and clinicians (Nishida et al., 2018). There was no comprehensive measure of substance use available for analysis to establish convergent validity. Finally, the IA for substance use operationalization used only two items and it is possible that operationalizations using more items could produce different findings.

This study tested an operationalization of a construct downstream from self-reflection and insight—IA. We focused on IA related to substance use and potential changes alongside symptoms over a year of treatment. Although the relationship of IA and an individual's illness management did not change over time, symptom remission did. Better IA for substance use was associated with greater symptoms, consistent with the insight paradox that past research showed in this population (Davis et al., 2020; Lysaker et al., 2018, Lysaker et al., 2007; Vohs et al., 2016). While this study addressed IA in a treatment context, the only specific treatment for IA tested in recent literature had a null finding and was pharmacological (Halverson et al., 2019), so psychosocial treatments targeting IA should be tested. In clinical practice in this population, feedback on IA could offer an inroad for clinicians to talk more openly with their clients about substance use during treatment. For clinicians, it could serve as a tool to conceptualize stages of change related to comorbid substance use treatment. Finally, in clinical research, the study of IA and substance use outcomes represents an opportunity for measurement-based care in assessing treatment response. Future work could evaluate this in medical databases, including comorbid substance use treatment in broader healthcare systems.

## Funding source

This study required no funding and was unsponsored.

## CRediT authorship contribution statement

*Joshua E. Mervis*: Conceptualization, Methodology, Formal analysis, Investigation, Writing – Original Draft, Writing - Review & Editing, Project administration.: *Jamie Fischer*: Writing – Review & Editing, Project administration.: *Samuel E. Cooper*: Formal analysis, Writing - Review & Editing.: *Andrew Colt Deckert*: Project administration.: *Paul H. Lysaker*: Writing - Review & Editing.: *Angus W. MacDonald, III*: Writing - Review & Editing.: *Piper Meyer-Kalos*: Conceptualization, Resources, Writing- Reviewing and Editing, Supervision.

## Declaration of competing interest

The authors declare that they have no conflicts of interest.
